# Supplementary material for: Use of Theory-Driven Report Back to Promote Lung Cancer Risk Reduction
Source: Int J Environ Res Public Health. 2021 Oct 11;18(20):10648. doi: 10.3390/ijerph182010648 (PMC8536114; doi:10.3390/ijerph182010648)
Supplement: Supplementary file 1 [file ijerph-18-10648-s001.zip › ijerph-1358113-supplementary.pdf]

FRESH Action Algorithm - Homeowners

Before beginning, make sure you have verified that the:

correct level of Radon/SHS is selected

Radon/SHS values are correct

correct Participant ID is entered

appropriate recruitment group is selected

As you go through the survey, please check the appropriate boxes for any items that you utilize during the interview. This survey contains "branching logic" which displays/hides information as items are selected/deselected. Once you have entered all information, you will need to submit the survey and complete the Qualtrics disposition. If you stop at any point during the survey, remember to press "save and return" and enter the appropriate validation code when you are ready to resume the survey (dispositions should be completed for partial interviews, also).

Participant ID # ________________________________

Date ________________________________

Are the radon and SHS levels high?

☐ No, Radon level is < 4 pCi/L
☐ Yes, Radon level is > 4, but < 8 pCi/L
☐ Yes, Radon level is > 8 pCi/L
☐ No, SHS level is < 0.1 ug/m3
☐ Yes, SHS level is > 0.1 ug/m3

This participant is a:

☐ Homeowner with Smoking in the Home
☐ Homeowner with NO Smoking in the Home

I am calling today to share the results of your radon and secondhand smoke tests, and discuss your thoughts about fixing your home. (Reinforce when you met at enrollment--mention the relationship/connection). Is this still a good time for you to talk? (If not, schedule another time to talk and complete disposition when you end the call noting the date/time of the next call in the comments.) This call may be recorded for quality assurance purposes.

[(Give good news first) If both radon and SHS are high, share radon first and discuss fully]

Your radon level was ________________________________
(pCi/L (picocuries per liter))

If radon level "< 4 pCiL"
Congratulations! Your home radon level is below the EPA action level, meaning it is within normal limits at this time. We encourage you to retest every two years. Radon test kits can be obtained from some local health departments or the state Radon Program.

If radon level "> 4 pCiL, but < 8 pCiL" OR "> 8 pCiL"
This level is above the Environmental Protection Agency suggested action level of 4 pCi/L.

Your secondhand smoke level was ________________________________
(ug/m3 (micrograms per cubic meter of air))

If SHS level "> 0.1 ug/m^3"
This level is considered a high level of secondhand smoke in the home (anything > 0.1 ug/m3 is considered high). The U.S. Surgeon General warns that even a little bit of secondhand smoke is hazardous.

If SHS level "< 0.1 ug/m^3"

Congratulations! Your level of nicotine in the air is within normal limits at this time. We encourage you to adopt a smoke-free policy in your home and vehicles if you have not already done so. We applaud you efforts to keep smoking outside of your home and away from windows, doorways, and vents.

ASSESS PAPM STAGE

RADON If radon level "> 4 pCiL, but < 8 pCiL" OR "> 8 pCiL"

What does it mean that your Radon is High? Radon is a colorless, odorless gas that exists naturally in the soil and rock beneath your home. It becomes a problem when it is trapped in your home. Living in a home with a radon level of 4 pCi/L is like getting 200 chest x-rays per year; a level of 20 pCi/L is like smoking two packs of cigarettes per day. It can be fixed. Mitigation is the term used for reducing radon in the home. A certified professional would install a pipe and fan system that reduces the radon levels. [Pause for response] (If appropriate, say "I think I may know the answers to the following questions, but I do not want to assume.")

Which of the following best describes your thoughts about radon mitigation for your home?

☐ I've never thought about mitigating (Stage 2)
☐ I'm undecided about mitigating (Stage 3)
☐ I've decided I don't want to mitigate (Stage 4)
☐ I've decided I do want to mitigate (Stage 5)

[As you are assessing PAPM Stage, you can say, 'Tell me your thoughts about that; What kind of things have you tried? (Where is the barrier-perception or failed attempts?)]

DETERMINE PAPM STAGE AND MOTIVATION TO TAKE ACTION A brief tailored problem solving approach will be based on the PAPM stage and individual needs or concerns to increase motivation and promote self-efficacy to take action.

USE MOTIVATIONAL INTERVIEWING PRINCIPLES THROUGHOUT Help them find the answers within themselves. They need to feel that they are coming to the decision on their own. If you find yourself giving them information without them requesting it, you are likely on the wrong track. o Instead of giving information per se, you can say, "People are often concerned about X" or "That is a reasonable concern." o 'It sounds like you are saying.....' (use reflection & summary statements) o Give information if they ask! • Do not 'sell'; you are Partnering. • Talk less, listen more • To help them make a decision, you can say: "Are there things you need to know to make a decision about ____? • Correct misperceptions in a gentle way. You can use self-disclosure (e.g., I just recently learned that COPD was a risk for lung cancer) but use it very sparingly. • Take a positive frame on their answers (see below under Important) • Use Persistent Redirection: "Good point. Would it be OK if we talk about X?" • Listen but redirect them to Stay on Message!

STRATEGIES TO INCREASE MOTIVATION TO TAKE ACTION

STAGE 2: I've never thought about mitigating (Relevance, Risks)

☐ Personalize threat relevance
☐ Engage social supports/social norms ('Have you talked with family/friends about this?')

If appropriate, you can assess how Important radon and/or SHS is to them.

☐ How do you feel at the moment about [insert topic]?
☐ How important is it to you personally to [insert topic]? If 0 was 'not important' and 10 was 'very important,' what number would you give yourself? Enter value below for "first" topic
☐ How do you feel at the moment about [insert topic]? How much do you want to [insert topic]? If 0 was 'not at all' and 10 was 'very much,' what number would you give yourself?Enter value below for "second" topic
☐ If they answer a low number, say, "How come it wasn't a '0'?"
☐ You can also say, "What makes it a 5?"

Importance rating for first topic ______________________

Importance rating for second topic ______________________

If appropriate, assess how Confident they are in taking action.

☐ If you decided right now to [insert topic] how confident do you feel about succeeding with this? If 0 was 'not confident' and 10 was 'very confident,' what number would you give yourself?

Confidence rating ______________________

If appropriate, assess how Ready they are to take action.

☐ If 0 was 'not ready' and 10 was 'ready,' what number would you give yourself?

Readiness rating ______________________________

STAGE 3: I'm undecided about mitigating (Relevance, Risks, Rewards, Roadblocks) ("Are there things you need to know to make your decision?)

☐ Explore perceived risk
☐ Explore perceived severity
☐ Explore perceived response efficacy ('How likely do you think mitigation will work?'-- Do they believe in the solution?)
☐ Explore perceived social norms ('What is happening in your neighborhood? ('Have any of your neighbors mitigated?')
☐ Explore affect (fear and worry; 'How scary is it that your levels are so high?')

If appropriate, you can assess how Important radon and/or SHS is to them.

☐ How do you feel at the moment about [insert topic]?
☐ How important is it to you personally to [insert topic]? If 0 was 'not important' and 10 was 'very important,' what number would you give yourself? Enter value below for "first" topic
☐ How do you feel at the moment about [insert topic]? How much do you want to [insert topic]? If 0 was 'not at all' and 10 was 'very much,' what number would you give yourself?Enter value below for "second" topic
☐ If they answer a low number, say, "How come it wasn't a '0'?"
☐ You can also say, "What makes it a 5?"?" or 'What is your X number based on?'

Importance rating for first topic ______________________________

Importance rating for second topic ______________________________

If appropriate, assess how Confident they are in taking action.

☐ If you decided right now to [insert topic] how confident do you feel about succeeding with this? If 0 was 'not confident' and 10 was 'very confident,' what number would you give yourself?

Confidence rating ______________________________

If appropriate, assess how Ready they are to take action.

☐ If 0 was 'not ready' and 10 was 'ready,' what number would you give yourself?

Readiness rating ______________________________

STAGE 4: I've decided I DON'T want to mitigate (Relevance, Risks, Rewards, Roadblocks)

☐ Explore decision processes ("Tell me how you came to your decision"); (Respect decision but make sure it's based on correct information)
☐ Explore decision pros/cons (Keep the conversation open! Say, 'Is it OK if we talk about the benefits of fixing your home for radon?')

If appropriate, you can assess how Important radon and/or SHS is to them.

Enter value below for "first" topic

☐ How do you feel at the moment about [insert topic]? How important is it to you personally to [insert topic]? If 0 was 'not important' and 10 was 'very important,' what number would you give yourself?
☐ How do you feel at the moment about [insert topic]? How much do you want to [insert topic]? If 0 was 'not at all' and 10 was 'very much,' what number would you give yourself? Enter value below for "second" topic
☐ If they answer a low number, say, "How come it wasn't a '0'?"
☐ You can also say, "What makes it a 5?"

Importance rating for first topic ______________________________

Importance rating for second topic ______________________________

If appropriate, assess how Confident they are in taking action.

☐ If you decided right now to [insert topic] how confident do you feel about succeeding with this? If 0 was 'not confident' and 10 was 'very confident,' what number would you give yourself?

Confidence rating __________________________________

If appropriate, assess how Ready they are to take action.

☐ If 0 was 'not ready' and 10 was 'ready,' what number would you give yourself?

Readiness rating __________________________________

STAGE 5: I've decided I DO want to mitigate (Repetition, Facilitate Mitigation Plan Development) ("Great decision. You have made a decision to protect your family. What is your next step?")

☐ Explore need to act
☐ Explore implementation plans (Explore what they need to do; Specific timeline that works for them; Specific plans; What assistance do they need to act?)
☐ Prepare reminders/cues
☐ Identify sources of assistance and potential barriers (What kinds of things may get in the way of you doing this?)

If radon level "> 4 pCiL, but < 8 pCiL"

Since your radon level is between 4 and 8 pCi/L, we recommend doing a follow-up long-term radon test to see if your radon levels change over time. A long-term test kit is placed in the home for at least 91 days. You can purchase a long-term kit for $25.95 at securechek.com/DIY/index.html. If your level still comes back at or above 4 pCi/L, we recommend fixing the problem. We will send you a voucher for $600 to help you with the cost of mitigation, whether you decide to fix your home now or later.

If radon level "> 8 pCiL"

Since your radon level is high, we will send you a voucher for $600 to help you with the cost of mitigation. The voucher includes a list of certified radon mitigators who will accept the study voucher for $600. If you would like to use another certified mitigator not related to the study, go to nrpp.info/radon_mitigation_service.shtml. Please note that the $600 voucher is good only with the two study-approved, certified radon mitigators.

If radon level "> 4 pCiL, but < 8 pCiL" OR "> 8 pCiL"

RELEVANCE

☐ Do you know anyone who has had problems from radon?
☐ Do people you know talk about it?
☐ Has anyone you know had lung cancer or died from it?
☐ Why is fixing your home for radon personally important to you?

Potential follow-up information tailored to participant response:

☐ Discuss personal health concerns
☐ Family health issues
☐ Length of time living in home

RISKS What do you see as the risks or negative aspects of radon exposure? Potential Follow-up Information Tailored to Participant Response: (only offer if they don't generate risks on their own)

☐ Although one cannot see or smell radon, exposure to this gas has a cumulative effect and may lead to lung cancer. Radon and secondhand smoke together in a home are a dangerous combination and it increases your risk for lung cancer. If you smoke or are exposed to secondhand smoke and your home has high levels of radon, the risk of getting lung cancer is especially high. You can easily reduce the levels of both secondhand smoke and radon in your home.

REWARDS What would be the pros of fixing your home for radon? Potential Follow-up Information Tailored to Participant Response:

☐ 'If you fix your home for radon, what do you think the impact will be on health, well-being, smell of house, sense of safety, risk?'
☐ We will supply you a voucher for up to $600 to help you cover the cost of mitigation. If you mitigate, this may be a benefit when you try to sell your home.
☐ Reduced health risk

If radon level "> 4 pCiL, but < 8 pCiL" OR "> 8 pCiL"

ROADBLOCKS What may be some reasons why you would choose not to fix your home for radon? (Cost, time energy, hassles, resale, social relationships) Potential Follow-up Information Tailored to Participant Response:

- ☐ Have you ever had a cost like this?; How did you handle it?; What are some things others have done to make home repairs?' REMEMBER TO STAY ON MESSAGE!
- ☐ Radon mitigation costs between $1500 and $2500 per home. As part of the study, we are providing a $600 voucher to help you with the cost.
- ☐ Some people think that radon is a hoax and don't think it's necessary to test or mitigate.
- ☐ Some people think all they have to do is seal cracks in the basement and open the windows. Mitigation by a certified mitigation professional is recommended to fix your home for radon.

If radon level "> 4 pCiL, but < 8 pCiL"

REPETITION STRATEGIES TO ENHANCE SELF-EFFICACY What might help motivate you to follow through with your plan to mitigate? Potential Follow-up Information Tailored to Participant Response:

- ☐ You can fix your home for high radon levels.
- ☐ Since your radon level is between 4 and 8 pCi/L, we recommend doing a follow-up long-term radon test to see if your radon levels change over time. A long-term test kit is placed in the home for at least 91 days. You can purchase a long-term kit for $25.95 at securechek.com/DIY/index.html. If your level still comes back at or above 4 pCi/L, we recommend fixing the problem. We will send you a voucher for $600 to help you with the cost of mitigation, whether you decide to fix your home now or later.

If radon level "> 8 pCiL"

REPETITION STRATEGIES TO ENHANCE SELF-EFFICACY What might help motivate you to follow through with your plan to mitigate? Potential Follow-up Information Tailored to Participant Response:

- ☐ You can fix your home for high radon levels.
- ☐ Since your radon level is high, we will send you a voucher for $600 to help you with the cost of mitigation. The voucher includes a list of certified radon mitigators who will accept the study voucher for $600. If you would like to use another certified mitigator not related to the study, go to nrpp.info/radon_mitigation_service.shtml . Please note that the $600 voucher is good only with the two study-approved, certified radon mitigators.

ASSESS PAPM STAGE

SECONDHAND SMOKE If SHS level "> 0.1 ug/m3"

What do you know about exposure to Secondhand Smoke? • Secondhand smoke contains 7,000 chemicals and nearly 70 are known to cause cancer. Secondhand smoke is just as dangerous as smoking. (You might say, 'a lot of people don't know this' if appropriate). (If they sound surprised, you could say 'Tell me what you know about SHS') • Limiting smoking to one room in the house does not protect your family, because the smoke travels easily through door cracks and ventilation systems. It can be easily fixed. Asking people to smoke outside, away from doors, windows, and vents, is the best way to protect your family. (If appropriate, say "I think I may know the answers to the following questions, but I do not want to assume.")

Which of the following best describes your thoughts about secondhand smoke in your home?

☐ I've never thought about asking people to smoke outside (Stage 2)
☐ I'm undecided about asking people to smoke outside (Stage 3)
☐ I've decided I don't want to ask people to smoke outside (Stage 4)
☐ I've decided I do want to ask people to smoke outside (Stage 5)
☐ I've implemented a no smoking policy in my home (Stage 6/7)

DETERMINE PAPM STAGE AND MOTIVATION TO TAKE ACTION A brief tailored problem solving approach will be based on the PAPM stage and individual needs or concerns to increase motivation and promote self-efficacy to take action.

USE MOTIVATIONAL INTERVIEWING PRINCIPLES THROUGHOUT Help them find the answers within themselves. They need to feel that they are coming to the decision on their own. If you find yourself giving them information without them requesting it, you are likely on the wrong track. o Instead of giving information per se, you can say, "People are often concerned about X" or "That is a reasonable concern." o 'It sounds like you are saying.....' (use reflection & summary statements) o Give information if they ask! • Do not 'sell'; you are Partnering. • Talk less, listen more • To help them make a decision, you can say: "Are there things you need to know to make a decision about ____? • Correct misperceptions in a gentle way. You can use self-disclosure (e.g., I just recently learned that COPD was a risk for lung cancer) but use it very sparingly. • Take a positive frame on their answers (see below under Important) • Use Persistent Redirection: "Good point. Would it be OK if we talk about X?" • Listen but redirect them to Stay on Message!

STAGE 2: I've never thought about asking people to smoke outside (Relevance, Risks)

☐ Personalize threat relevance
☐ Engage social supports/social norms

If you haven't signed a smoke-free home pledge or posted a no-smoking sign, now would be a good time to consider that. A smoke-free home pledge or no-smoking sign is simply a formal way to asks family members and visitors to smoke outside, without exceptions. Please also ask them to smoke at least 20 feet away from doors, windows, and vents to prevent the smoke from entering the house.

STAGE 3: I'm undecided about asking people to smoke outside (Relevance, Risks, Rewards, Roadblocks)

☐ Explore perceived risk
☐ Explore perceived severity
☐ Explore perceived response efficacy
☐ Explore perceived social norms
☐ Explore affect (fear and worry)

If you haven't signed a smoke-free home pledge or posted a no-smoking sign, now would be a good time to consider that. A smoke-free home pledge or no-smoking sign is simply a formal way to asks family members and visitors to smoke outside, without exceptions. Please also ask them to smoke at least 20 feet away from doors, windows, and vents to prevent the smoke from entering the house.

STAGE 4: I've decided I don't want to ask people to smoke outside (Relevance, Risks, Rewards, Roadblocks)

☐ Explore decision pros/cons
☐ Explore decision processes

STAGE 5: I've decided I do want to ask people to smoke outside (Repetition, Facilitate Mitigation Plan Development)

☐ Explore need to act (You might say, 'What do you think might reduce your levels?')
☐ Explore implementation plans [You might say, 'What do you think would be the specifics of implementing a plan?;]
☐ Explore any expected barriers
☐ Prepare reminders/cues
☐ Identify sources of assistance

If you haven't signed a smoke-free home pledge or posted a no-smoking sign, now would be a good time to consider that. A smoke-free home pledge or no-smoking sign is simply a formal way to asks family members and visitors to smoke outside, without exceptions. Please also ask them to smoke at least 20 feet away from doors, windows, and vents to prevent the smoke from entering the house.

STAGE 6/7: I've implemented a no smoking policy in my home (Reinforce Change, Relapse Prevention)

☐ Congratulate and support no smoking policy in the home
☐ Discuss ways of communicating the policy to family, visitors
☐ Explore thoughts about maintaining dedication to the plan
☐ Identify potential challenges or barriers and ways to prevent relapse

If you haven't signed a smoke-free home pledge or posted a no-smoking sign, now would be a good time to consider that. A smoke-free home pledge or no-smoking sign is simply a formal way to asks family members and visitors to smoke outside, without exceptions. Please also ask them to smoke at least 20 feet away from doors, windows, and vents to prevent the smoke from entering the house.

RELEVANCE

☐ Do you know anyone who has had problems from secondhand smoke?
☐ Do people you know talk about it?
☐ Has anyone you know had lung cancer or died from it?

Potential Follow-up Information Tailored to Participant Response:

☐ Discuss health concerns, family issues, and length of time living in home, exposure in workplace and public places.

RISKS What do you see as the risks of secondhand smoke exposure? Potential Follow-up Information Tailored to Participant Response:

☐ Even if people do not smoke inside, secondhand smoke can still get in the house. The U.S. Surgeon General says there is no risk-free level of secondhand smoke. Secondhand smoke and radon are a dangerous combination. By not fixing your home for radon and secondhand smoke, you are putting you and your family members at risk for lung cancer, premature death, and other diseases.

REWARDS Question: What would be the pros of fixing your home for secondhand smoke? Potential Follow-up Information Tailored to Participant Response:

☐ Going smoke-free is a benefit when you try to sell your home.
☐ Sometimes when you make your home smoke-free, the smoker in the house may be interested in quitting smoking. You can call the Quitline (1-800-QUITNOW) to get more information about how to quit smoking and/or help a family member or significant other to quit. The most important thing a smoker can do for their health is to quit, and there are ways to help them when they are ready.
☐ To make it easier to make your home smoke-free, we gave you a magnetic smoke-free home picture frame when we enrolled you in the study. Place a picture of a loved one in the frame and put it on your refrigerator as a reminder!

ROADBLOCKS What may be some reasons why you would choose not to make your home smoke-free? Potential Follow-up Information Tailored to Participant Response:

☐ If you have secondhand smoke in your home, short-term radon test results may be artificially low because radon gas attaches to smoke particles.
☐ Move ashtrays away from the doorways to encourage family members and visitors to not smoke in the home or close to the doorways.
☐ Ask smokers to cover clothing with a jacket when outside smoking; leave jacket outside when returning to the home.

REPETITION STRATEGIES TO ENHANCE SELF-EFFICACY What might help motivate you to follow through with your plan to make your home smoke-free? Potential Follow-up Information Tailored to Participant Response:

☐ Signing the smoke-free home pledge is an easy way to show that you are protecting your family from secondhand smoke in and around your home (we will mail you one!). You can also protect your family by avoiding secondhand smoke in public places - limiting exposure in all areas of your life is a good idea.

CONCLUDING THE INTERVIEW

SESSION CONCLUSION

- ☐ 1) Provide a summary of the discussion regarding radon mitigation and secondhand smoke reduction plan.
- ☐ 2) Offer to clarify or correct any misunderstandings from the summary
- ☐ 3) Is there other info that you need?
- ☐ 4) Reminder that we will mail materials to help
- ☐ 5) Reminder we'll have a few more follow-up surveys and then will ask them to test again at the end
- ☐ 6) Verify their address and all contact information. Make any updates at http://tinyurl.com/bkgttdl.YOU MUST PRESS AND HOLD CTRL WHEN CLICKING THE LINK TO OPEN THE QUALTRICS TAB IN A NEW WINDOW.
- ☐ 7) Thank them for taking part in FRESH and End the call

Comments made by the Participant or Notes about the Conversation

Actions or follow-ups needed by Staff
